# An Updated Evaluation of Intrathecal IgG Synthesis Markers in Relation to Oligoclonal Bands

**DOI:** 10.3390/diagnostics13030389

**Published:** 2023-01-20

**Authors:** Fotini Boufidou, Aigli G. Vakrakou, Maria Anagnostouli, Kostas Patas, Georgios Paraskevas, Stylianos Chatzipanagiotou, Leonidas Stefanis, Maria-Eleftheria Evangelopoulos

**Affiliations:** 1 Neurochemistry and Biological Markers Unit, 1st Department of Neurology, Eginition Hospital, Medical School, National and Kapodistrian University of Athens, 115 28 Athens, Greece; 2 Department of Biopathology, Eginition Hospital, Medical School, National and Kapodistrian University of Athens, 115 28 Athens, Greece; 3 1st Department of Neurology, Eginition Hospital, Medical School, National and Kapodistrian University of Athens, 115 28 Athens, Greece; 4 2nd Department of Neurology, Attikon University Hospital, Medical School, National and Kapodistrian University of Athens, 12462 Athens, Greece

**Keywords:** intrathecal synthesis markers, oligoclonal bands (OCBs), hyperbolic function, multiple sclerosis (MS), cerebrospinal fluid (CSF) analysis, blood-derived CSF molecules

## Abstract

The aim was to evaluate the performance of the latest quantitative marker for intrathecal IgG synthesis and to compare it with other established markers used for the same purpose. We retrospectively applied Auer’s and Reiber’s intrathecal IgG synthesis formulae in a cohort of 372 patients under investigation for central nervous system demyelination who had undergone lumbar puncture and oligoclonal bands (OCBs) detection for demonstrating intrathecal IgG synthesis. A ROC analysis revealed Auer’s formula had lower sensitivity (68%) compared to Reiber’s formula (83%) and IgG index (89%), in our cohort of patients that exhibited normal to mildly elevated albumin quotients (4.48 ± 3.93). By excluding possible sources of errors, we assume that Auer’s formula is less sensitive than other established tools for the “prediction” of the detection of OCBs in routine cerebrospinal fluid (CSF) analyses due to the mathematical model used. Given the ability of Reiber’s hyperbolic formula to describe the blood–CSF IgG distribution across a wide range of blood–brain barrier functionality, its use and the use of similar formulae are recommended for the discrimination between CNS-derived and blood-derived molecules in clinical laboratories.

## 1. Introduction

The demonstration of intrathecal immunoglobulin synthesis (ITIS) as a part of the immune process within the central nervous system (CNS) has always been of great diagnostic significance and a major diagnostic challenge for all methodological developments in cerebrospinal fluid (CSF) analysis. For the qualitative demonstration of ITIS, isoelectric focusing (IEF) coupled with immunodetection (immunoblotting or immunofixation) has become the leading technique for the detection and study of oligoclonal bands (OCBs) in CSF [1]. In addition to qualitative measures, the quantitative interpretation of ITIS has been a methodological challenge and several formulae have been proposed and evaluated for this purpose [2]. Among them, the linear IgG index [3,4] has been broadly used in the past, mainly because of its simplicity, but it has been replaced by non-linear formulae, such as Reiber’s hyperbolic formula [5,6,7] as they better reflect human neurophysiology. However, all ITIS formulae lack sensitivity and specificity compared to IEF [2,6,8], and any improvement would certainly optimize diagnostic accuracy. Therefore, the introduction of a new empirical formula for the quantification of ITIS by Auer et al. was viewed as a promising new approach for a new diagnostic tool aiming at fewer false-positive results than Reiber’s formula [9]. Moreover, a major progress in this field has been the quantification of immunoglobulin light chains, which are secreted as B-cells’ by-products, i.e., free light chains (FLC) [10]. FLC have been reported to be increased in MS (Multiple Sclerosis) CSF [11] and are now proposed to be a quantitative biomarker for the disease. Reference values and the debate regarding the optimal diagnostic use of intrathecal FLC synthesis are close to a final consensus.

From this perspective, we conducted this study to evaluate the new formula by Auer et al. for IgG in a clinical cohort considering OCBs as the reference method for the first time and to compare the performances of different kinds of quantitative ITIS markers.

## 2. Materials and Methods

### 2.1. Study Cohort

To perform this retrospective study, we collected data from a laboratory computerized database for patients who have been hospitalized between 2015 and 2017 in the Demyelinating Diseases Unit of the Neurology Department of Eginition University Hospital. All patients enrolled in the study had imaging features suggestive of demyelination with or without clinical manifestations and underwent diagnostic lumbar puncture. Most of them were in stable condition during sample collection and drug naïve. Diagnosis of MS was in accordance with McDonald2017 criteria [1]. The clinically isolated syndrome (CIS) group included patients who had had a first demyelinating episode, with clinically monofocal or multifocal presentations and at least one asymptomatic lesion identified by magnetic resonance imaging (MRI) [12].From the initial group of 387 patients, 5 had missing data in basic CSF analysis and 10 were considered to have blood-contaminated CSF samples (>2000 red blood cells/μL). These 15 patients were excluded and finally 372 patients participated in the study.

### 2.2. Biological Samples and Assays

After overnight fasting, a CSF and a blood sample were collected between 8.00 and 10.00 in the morning by lumbar and venous puncture, respectively. The maximum time interval between them was one hour and during that no major therapeutic interventions occurred that could affect measurements of both biological fluids (infusions, transfusions, immune-modulating medication). CSF red- and white-blood-cell concentrations were estimated using a Fuchs–Rosenthal chamber and this procedure was completed within two hours after lumbar puncture. Afterwards, CSF and blood samples were centrifuged at 2000× *g* for 10 min at room temperature and part of the supernatants was used for the nephelometric determination of IgG and albumin (alb) levels (BN II system, Siemens Healthcare GmbH, Erlangen, Germany). Serum and CSF aliquots from the rest of the supernatant were kept frozen in −20 °C for further analyses.

OCB detection by IEF on agarose gel was performed on the semi-automatic HYDRASYS system within a maximum of one week after lumbar puncture. Initially, CSF and serum IgG concentrations were adjusted to each other. Then, isofocusing with the use of Hydragel 9 CSF isofocusing kit followed by immunofixation with the use of antiserum anti-IgG conjugated to peroxidase (SEBIA, Evry Cedex, France) were performed. Recognized positive and negative controls ran with each set of samples. CSF findings were directly compared with serum samples’ findings, running simultaneously in the same assay in an adjacent track. Finally, the IgG immunodetection patterns of CSF and serum from the same patient were visually compared and categorized into five OCB constellation types (I–V), according to the European consensus paper of 1994 [13]. Type II and type III patterns were classified as positive for intrathecal IgG synthesis and types I, IV, and V were classified as negative. At least two additional bands in CSF compared to serum were considered necessary to characterize a CSF sample as OCB positive (type II or III). Two experienced medical laboratory doctors conducted the evaluation.

For the quantitative determination of intrathecal IgG synthesis, the CSF/serum quotients (QIgG and Qalb) and the IgG index (QIgG/Qalb) were calculated. Samples with QIgG/Qalb ≥ 0.65 were considered positive for intrathecal IgG synthesis [3,4]. For this study’s purposes, two more quantitative ITIS estimates using different upper limits for QIgG were retrospectively calculated, i.e., Reiber’s hyperbolic formula (Qlim = 0.93√(Qalb^2^ + 6 × 10^−6^) −1.7 × 10^−3^) [5] and formula by Auer et al. (Qlim = 0.882 × Qalb^1.035^) [9].

### 2.3. Ethics Approval

Written informed consent was obtained from the study participants and the study was approved by the Ethics Committee of Eginition Hospital, Athens University Medical School (501/30 July 2019), in accordance with the current version of the Declaration of Helsinki.

### 2.4. Statistical Analysis

Statistical analysis and the graphical representation of the data were performed using GraphPad Prism (version 6.0, GraphPad Software, San Diego, CA, USA). Receiver operating characteristic (ROC) curve analysis, which calculated the area under the ROC curve (AUC), was used to determine the diagnostic accuracy of IgG index, Reiber’s IgG formula, and Auer’s IgG formula as markers for increased probability of finding a positive OCB result in CSF analysis, indicative of intrathecal synthesis. The positive predictive value (PPV) was calculated as [true-positive/(true-positive + false-positive)], and the negative predictive value (NPV) as [true-negative/(true-negative + false-negative)], with reference positive values in the presence of OCB.

Classification accuracy (ACC) was calculated as follows: True Positive + True Negative/True Positive + True Negative + False Positive + False Negative. Using a 2 × 2 traditional confusion matrix for binary classifications, Cohen’s kappa formula was calculated as follows: κ = 2 × (TP × TN−FN × FP)/[(TP + FP) × (FP + TN) + (TP + FN) × (FN + TN)], where TP is true-positive, FP is false-positive, TN is true-negative, and FN is false-negative. Cohen’s kappa minimum value is −1 (wrong prediction) and the maximum value is +1 (perfect classification). Furthermore, if κ ≈ 0, the prediction made was similar to random prediction [14].

## 3. Results

### 3.1. CSF Analysis Measurements 

A total of 372 neurological inpatients were included in the analysis. The clinical and demographic characteristics of the patients are presented in Table 1. The CSF analysis parameters are presented in Table 2.

### 3.2. Classification Performance of Different Formulae, IgG Index, Auer and Reiber’s Formulae for the Presence of OCB Indicative of Intrathecal Synthesis

We performed an ROC analysis at the cut-off levels described in the Materials and Method section. The sensitivity, specificity, and positive and negative predictive values of all the investigated parameters are given in Table 3.

**Figure 1:**
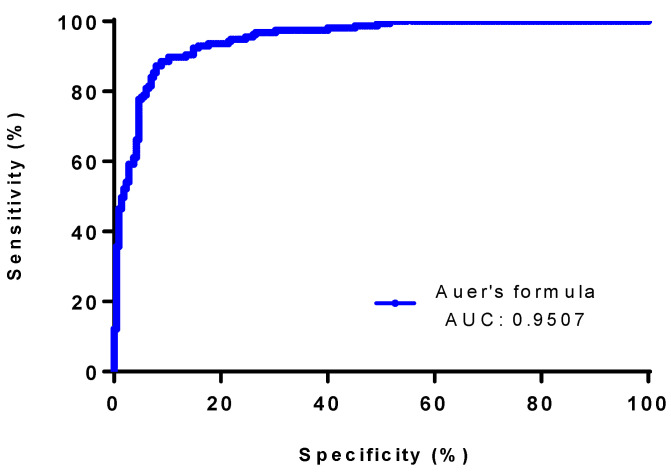
The ROC curve of Auer’s et al. formula for discriminating between OCB-positive (n = 157) and negative (n = 215) patients. Abbreviations: AUC, area under the curve.

**Figure 2:**
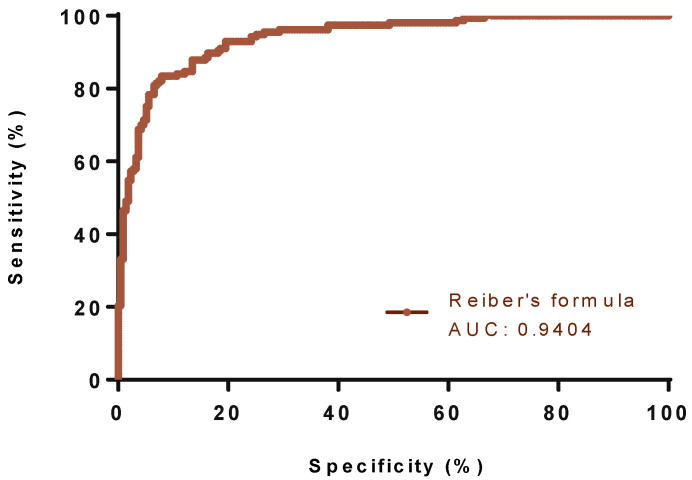
The ROC curve of Reiber’s formula for discriminating between OCB-positive (n = 157) and negative (n = 215) patients. Abbreviations: AUC, area under the curve.

**Figure 3:**
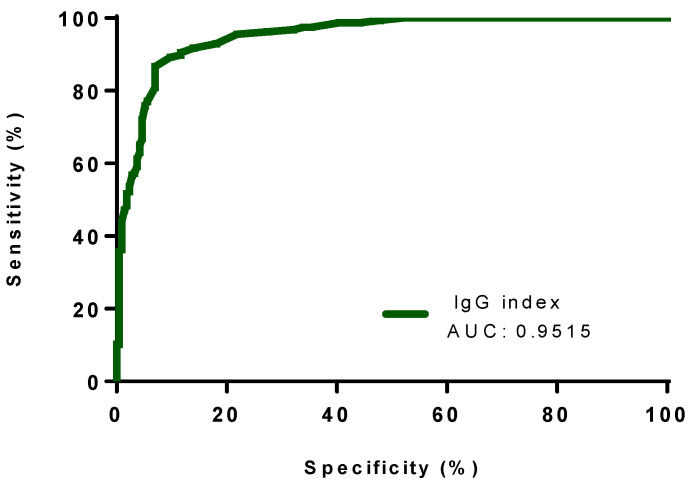
The ROC curve of the IgG index for the discrimination between OCB-positive (n = 157) and negative (n = 215) patients. Abbreviations: AUC, area under the curve.

To distinguish OCB-positive (for intrathecal synthesis) patients from OCB-negative patients, the IgG index showed the highest AUC with a value of 0.9515 (95% CI; 0.9312 to 0.9718), as compared with the other quantitative formulae. Reiber and Auer’s formulae exhibited AUC levels of 0.9404 (95% CI; 0.9171 to 0.9636) and 0.9507 (95% CI; 0.9302 to 0.9712), respectively (Table 3, Figure 1, Figure 2 and Figure 3).

Based on past experience, we applied cut-off levels of 0.65 for the IgG index and we found it had the highest sensitivity (89%) in verifying positive OCB results, albeit with lower specificity (90%) compared to the other two formulae (specificity Reiber’s and Auer’s formulae: 92 and 95%, respectively). For the detection of OCBs, cut-off levels of >0.93 for Reiber’s formula and >0.882 for Auer’s formula were used, resulting in sensitivities that showed the superiority of Reiber’s formula (83%) compared to Auer’s formula (68%) (Table 3). Auer’s formula yielded false-negative results in 52 out of the 157 OCB-positive patients in our cohort, scoring the lowest NPV (81%).

### 3.3. Accuracy Rates of Different Formulae, IgG Index, and Auer’s and Reiber’s Formulae for the Presence of OCB Indicative of Intrathecal Synthesis

As the ROC curves among the different formulae (IgG index, Reiber’s formula, and formula by Auer) were similar, we applied further statistical tests and metrics to better measure each method’s classification performance. To assess the accuracy rate, we used the proposed cut-off levels by Auer (>0.88) and Reiber (>0.93) and the standard cut-off level for the IgG index that had already been validated by our lab (>0.65) (Table 3). We calculated the classification accuracy (ACC) for the three formulae, defined as a ratio between correctly classified samples (positive and negative classes) and the total number of input samples. The IgG index resulted in the highest accuracy with a value of 0.898, meaning that by applying it, we could achieve predictions that were approximately 90% correct from a total of 100 measures (Table 3). Between Reiber’s and Auer’s formulae, the former displayed a higher accuracy with a value of 0.885, compared to the latter with a value of 0.839. Cohen’s kappa was further applied to assess the performance of the classification models for the used qualitative measures. It was chosen as it is a metric that additionally considers the possibility of the agreement of methods occurring by chance. The best k value among the three methods was again achieved by the IgG index, which showed comparable κ with that of Reiber’s formula, whereas the formula by Auer showed worse classification measures (Table 3).

## 4. Discussion

In 2016, Auer et al. [9] introduced three variations of a new empirical formula for the quantification of ITIS: one for each of the three main immunoglobulin subclasses (IgG, IgM, IgA). We retrospectively evaluated the IgG formula in a cohort of patients with suspected demyelinating disease, considering OCBs as the reference method, and compared it with other established quantitative ITIS markers of different kinds.

Although ITIS is found in various, mainly inflammatory CNS diseases [15], it reaches its maximal value in the differential diagnosis of MS. Indeed, despite repeated revisions of the diagnostic criteria for MS over the past two decades [16,17,18], the detection of OCBs in CSF remains one of the laboratory criteria supporting the clinical diagnosis of MS. Moreover, the fact that a positive result predicts the conversion of clinically isolated syndrome (CIS) to MS has been appreciated by many [19,20], and thus, in the latest revision, the value of the test was re-emphasized [1].

Nowadays, agarose IEF methods combined with immunodetection (immuno-fixation or immune-blotting) to increase detection sensitivity and provide a high specificity have been proposed for detecting IgG OCBs in the human CSF in diagnostic laboratories [1,21]. However, IEF followed by immunodetection, even when performed under strict rules [6], exhibits several methodological problems [7]. The lack of objectivity in interpreting the results is the most crucial among them. Therefore, the need for a complementary, objective diagnostic tool that could detect ITIS quantitatively in the form of a mathematical formula is of great importance and the goal of several scientific efforts. Indeed, reliable formulae exhibit several advantages over IEF as they use standardized determinations produced by automated analytical methods, such as nephelometry in the form of CSF/serum quotients, which finally give method-independent results that can be used over the course of the disease and also in multicenter studies. In 1972, the first formula of this kind was introduced by Delpech and Lichtblau [3]. Since then, numerous attempts have been made to imprint the linear or non-linear correlation between immunoglobulin and albumin CSF/serum quotients in other formulae [4,8,22,23,24,25,26]. Despite these efforts, all ITIS quantitative methodologies lack sensitivity and specificity compared to IEF [2,6,8]. Consequently, the introduction of new formulae and improvements in previous formulae are important. In this study, we applied Auer’s new formula along with Reiber’s formula for the IgG and IgG index in a cohort of 215 OCB-negative and 157 OCB-positive patients. Then, we performed a ROC curve analysis to compare the performance characteristics between these three formulae. Regarding specificity, some degree of superiority was revealed for Auer’s formula over Reiber’s formula and the IgG index (95% over 92% and 90%, respectively). On the contrary, the new formula had a low sensitivity (68%). This performance resulted from 52 false-negative patients, according to Auer’s formula, out of the 157 OCB-positive patients in our cohort. Among them, 29 patients were true-positive according to the other two formulae. The clinical diagnoses for those patients were radiologically isolated syndrome (RIS) (3/29), CIS (8/29), relapsing–remitting MS (RRMS) (12/29), and primary progressive MS (PPMS) (6/29). The sensitivity rate of Auer’s formula was considerably lower than that of Reiber’s formula (83%). Low sensitivity in these cases may significantly affect the final laboratory result, especially when IEF reveals “faint” bands or an ambiguous finding. A “negative” ITIS marker may prevent laboratory professionals from repeating IEF and finally lead to a false-negative result for this rater-dependent method and a bad performance for the selected ITIS marker complementary to IEF.

The IgG index, exhibiting a 89% sensitivity rate and 90% specificity rate, performed quite well in this particular cohort of patients with normal to mildly elevated QAlb (4.38 ± 3.93). However, it is well known that, due to its linearity, it can be proved unreliable in patients with severe blood–brain barrier (BBB) dysfunction and high QAlb in contrast to Reiber’s formula. Similarly, our results showing 68% sensitivity for Auer’s formula cannot be extrapolated to patients with other diseases than demyelinating diseases, particularly to patients with moderate-to-severe BBB dysfunction.

To our knowledge, this is the first and only evaluation of this kind for Auer’s et al. formula, besides the initial one [9]. A different type of evaluation for this formula based on clinical diagnoses was performed by the same group that introduced it in a cohort of patients with CIS and MS, independently of their OCB status [27]. They found lower sensitivity for Auer’s (65%) compared to Reiber’s formula (68%), a difference consistent with our findings. Our study was designed from a different perspective, and agrees with other researchers in this field who have advocated for the necessity of “the comparison of the analytical performances of Auer’s et al. formula with those of OCBs which remains the best way to assess intrathecal IgG production” [7].

Since the calculation of the new formula was based on nephelometric determination in CSF and serum from OCB-negative patients, a possible explanation of the new formula’s failure in “predicting” OCB positivity could be a methodological error in the techniques used. Nephelometric determination used in both studies is standardized and the use of CSF/serum quotients eliminates any possible inaccuracy, almost excluding them from being the source of error. Regarding the detection of OCBs, Auer et al. used IEF and subsequent immunoblotting according to Keir et al. with the modification of using polyacrylamide instead of agarose gel. The same technique has been repeatedly proposed as the “gold standard” for detecting the presence of OCBs in CSF [6,7,28,29,30]. We also used IEF followed by immune fixation instead of immunoblotting, which is also a leading technique [1]. Thus, the possibility that the difference between the immune-detection methods could have been the cause of discrepancies seems unreasonable.

A final possible source of error is the mathematical model that was used for describing a biophysical model. The IgG index describes the correlation between QIgG and Qalb as a linear function and ignores the nonlinear relationship between two blood-derived molecules of different sizes [5]. Auer et al.’s formula, due to its inherent ability to act as a non-linear molecular size-dependent simple power function, does not rely on the CSF flow rate. In contrast, Reiber’s hyperbolic function incorporates molecule diffusion across both the BBB and CSF flow rates [31]. By revealing Reiber’s hyperbolic function superiority over the simple power function, this study supports the use of similar models aiming to determine the intrathecal synthesis of immunoglobulins but also other molecules in clinical settings. Indeed, the field of markers for ITIS has attracted recent interest after the application of new, fully automated nephelometric and turbidometric assays [32] which enable the non-laborious and reliable quantification of a free immunoglobulin component, which is produced in excess during immunoglobulin syntheses, i.e., FLC [10,11]. FLC have been reported to be increased in MS CSF and since then, scientific efforts have focused on incorporating FLC CSF and blood levels, especially of the kappa isotype (kappa free light chains; KFLC), alone or appropriately correlated with markers of (BBB) permeability [33,34] in routine CSF analyses as an alternative quantitative marker for ITIS with a high sensitivity and specificity for MS [34,35,36,37,38]. The specific biophysical background and the technical particularities that have to be faced concerning this complex issue have been extensively described, and a theoretically and empirically founded hyperbolic function for FLC in correspondence to former schemes for IgG, IgM, and IgA immunoglobulins was introduced by Reiber in 2019 [39]. By using this scheme for KFLC as a screening tool, Hannich et al. proposed a laboratory workflow that may reasonably exclude a considerable number of CSF samples from further analyses, thus limiting unnecessary detections of OCBs and improving diagnostic accuracy [40]. By supporting the superiority of a hyperbolic function for the determination of intrathecal humoral immune responses, the findings of this study might contribute to the broader use of such novel workflows for the improvement of daily clinical practice.

## 5. Conclusions

Using IEF followed by immune fixation as our reference method, Auer’s et al. simple power function for IgG, although scoring high in specificity (95%), lacked sensitivity (68%) in patients with demyelinating diseases. Our data revealed the superiority of Reiber’s hyperbolic function over it, exhibiting a 83% sensitivity. The IgG index performed quite well in our cohort of patients with normal to slightly impaired BBB, and it can be used as an additional linear formula in these cases. Efforts to introduce an ITIS estimate of the new promising KFLC molecule are in progress and close to a final consensus. Our results favor the use of a hyperbolic function for the optimal discrimination between blood-derived and CNS-derived CSF molecules, such as KFLC, within a broad range of CSF functionality.

## Figures and Tables

**Table 1:** Clinical and demographic characteristics of all 372 patients.

Gender	No (%)
Female	225 (60.5)
Male	147 (39.5)
Age (mean ± SD)	38 years (12)
Final Diagnosis	No (%)
RRMS	204 (54.8)
CIS	141(38.0)
PPMS	19 (5.2)
RIS	8 (2.0)

RRMS, relapsing–remitting multiple sclerosis; CIS, clinically isolated syndrome; PPMS, primary progressive multiple sclerosis; RIS, radiologically isolated syndrome.

**Table 2:** Cerebrospinal fluid analysis of 372 MS patients.

CSF Parameters	Median ± SD
White Blood Cell (/μL)	2 (11.15)
Red Blood Cell (/μL)	2 (190)
CSF IgG (mg/dL)	39 (21.99)
QAlb	4.48 (3.932)
QIgG	3.08 (3.109)
OCBs	No (%)
Negative OCBs pattern 1	208 (55.9)
Positive OCBs pattern 2	155 (41.7)
Positive OCBs pattern 3	2 (0.5)
Negative OCBs pattern 4	7 (1.9)

OCB, oligoclonal bands; CSF, cerebrospinal fluid; Alb, albumin; IgG, immunoglobulin of IgG isotype.

**Table 3:** Results of ROC analysis.

Formula	Cut-Off	Sensitivity	Specificity	AUC (95% CI)	Std. Error	*p* Value	PPV (%)	NPV (%)	ACC	κ
IgG index	>0.65	0.89 (0.83–0.94)	0.9023 (0.86–0.94)	0.9515 (0.9312 to 0.9718)	0.01	<0.0001	86.96	91.94	0.898	0.792
Reiber’s formula	>0.93	0.83 (0.76–0.88)	0.92 (0.88–0.95)	0.9404 (0.9171 to 0.9636)	0.01	<0.0001	87.33	88.29	0.885	0.762
Auer’s formula	>0.88	0.68 (0.60–0.75)	0.9535 (0.92–0.98)	0.9507 (0.9302 to 0.9712)	0.01	<0.0001	91.59	81.02	0.839	0.658

ROC, receiver operating characteristics; AUC, area under the curve; PPV, positive predictive value; NPV, negative predictive value; CI, Confidence Interval; Classification Accuracy; κ, Cohen’s Kappa Coefficient. The cut-off values for the ROC curves are derived from the literature [5,9].

## Data Availability

Data supporting our findings can be found in the Tables. Additional data extracted may be shared upon request.
